# Still No Evidence for an Effect of the Proportion of Non-Native Speakers on Natural Language Complexity

**DOI:** 10.3390/e26110993

**Published:** 2024-11-18

**Authors:** Alexander Koplenig

**Affiliations:** Department of Lexical Studies, Leibniz Institute for the German Language (IDS), 68161 Mannheim, Germany; koplenig@ids-mannheim.de

**Keywords:** language complexity, language models, linguistic niche hypothesis, language typology, non-native speakers, quantitative linguistics

## Abstract

In a recent study, I demonstrated that large numbers of L2 (second language) speakers do not appear to influence the morphological or information-theoretic complexity of natural languages. This paper has three primary aims: First, I address recent criticisms of my analyses, showing that the points raised by my critics were already explicitly considered and analysed in my original work. Furthermore, I show that the proposed alternative analyses fail to withstand detailed examination. Second, I introduce new data on the information-theoretic complexity of natural languages, with the estimates derived from various language models—ranging from simple statistical models to advanced neural networks—based on a database of 40 multilingual text collections that represent a wide range of text types. Third, I re-analyse the information-theoretic and morphological complexity data using novel methods that better account for model uncertainty in parameter estimation, as well as the genealogical relatedness and geographic proximity of languages. In line with my earlier findings, the results show no evidence that large numbers of L2 speakers have an effect on natural language complexity.

## 1. Introduction

The linguistic niche hypothesis proposes that the social niche a language occupies in a community affects its structural properties. Specifically, according to the linguistic niche hypothesis, languages with large numbers of speakers tend to simplify their grammar and have a reduced structural complexity [1,2].

The linguistic niche hypothesis assumes that languages that are spoken by more people over greater geographic areas will, on average, also be learned by a greater proportion of L2 learners, i.e., often adults. Since complex morphology appears to be difficult to learn for adults, the linguistic niche hypothesis conjectures that there should be a negative selection over time against such hard-to-learn morphological paradigms for languages with a larger number of L2 speakers compared to languages that are mainly learned during childhood as L1 (first language). This, in turn, it is argued, explains the observed negative statistical association between speaker population size and morphological complexity [2,3]. In a recent study published in *Royal Society Open Science* [4], I have pointed out that since the argument outlined above is inductive by nature, its validity cannot be simply taken (more or less implicitly) for granted. Crucially, Lupyan and Dale [1] use the estimated speaker population size as a proxy for the proportion of L2 speakers [5]. In my paper [4], I tested this conjecture empirically for more than 2000 languages and showed that the results question the idea of the impact of non-native speakers on the grammatical and statistical structure of languages.

The main obstacle in this context is the fact that, as [6] points out, estimations regarding a breakdown of L1 versus L2 populations are very limited. In general, most information regarding speaker population sizes/compositions is based on *Ethnologue* [7], the most comprehensive and most widely consulted catalogue of languages that provides information and statistics for the languages of the world [8,9]. *Ethnologue* categorises each language in regard to how endangered it is using the Expanded Graded Intergenerational Disruption Scale (EGIDS) [10]. In this context, a language is categorised as vehicular if it is used as an L2 in addition to being used as an L1.

This information can be used to indirectly gain information about the proportion of L2 users: “A language at EGIDS 4 or lower is, by definition, a local language and L2 users are not expected. However, languages at EGIDS 3 and higher are vehicular and, by definition, they should have a significant number of L2 users” [7]. The great advantage here is that information at the EGIDS level is available for all languages that are listed in the Ethnologue. Table 1 provides an overview of each EGIDS level together with the mapping to vehicularity, a corresponding description taken from [10] and example languages taken from my dataset. In my paper, I used vehicularity as an indicator of whether a language is used by a large proportion of L2 speakers or not in order to test the assumed relationship between the L2 proportion and (morphological/information-theoretic) complexity. Through a series of statistical analyses, I tried to show that large L2 proportions do not seem to affect the (grammatical or information-theoretic) complexity of a language.

In a recent comment published in the *Journal of Language Evolution*, Kauhanen, Einhaus and Walkden ([11]; KEW) challenge my findings. KEW criticised both the use of vehicularity as a (binary) indicator of whether a language has a significant number of L2 users and the idea of imputing a zero proportion of L2 speakers to non-vehicular languages whenever a direct estimate of that proportion is unavailable. While I recognise the importance of post-publication commentary on published research, I will show in this paper that both points of criticism are explicitly mentioned and analysed in my paper. In addition, I will also comment on other points raised by KEW and demonstrate that both alternative analyses offered by KEW do not stand up to closer scrutiny.

However, I agree with KEW that a further study using both better data and better methods would certainly be desirable since testing for a link between language and social structure is turning out to be more complex than I once thought, as recently summarised in an excellent review on the subject [12].

In my original paper, information-theoretic complexity was estimated with a comparatively simple non-parametric statistical entropy estimation method [13] and was solely based on a very peculiar text type, i.e., parallel translations of the Bible, and there are several important challenges that the use of the Bible as a parallel text source pose [14,15,16]. Additionally, complexity was measured only on the level of characters, which is problematic due to cross-linguistic differences in the mapping between phonemes and graphemes [17,18]. This paper addresses these points by adding new data on the information-theoretic complexity of languages, with estimates derived at the levels of characters, words and sub-word units using an ensemble of different language models, ranging from simple statistical models to advanced neural networks. Estimates are based on a database of 40 multilingual text collections comprising a wide variety of text types, including nearly 1.5 billion words across more than 3700 documents in over 1100 languages [19,20].

With respect to improved methods, I re-analysed the novel information-theoretic complexity data using a frequentist multi-level multi-model averaging approach [20,21], which accounts for model uncertainty related to language- and document-specific characteristics when estimating model parameters. Additionally, to better control for the genealogical and geographic relatedness of languages, I re-analysed the morphological complexity data using an approach that combines spatial autoregressive modelling [22] with frequentist multi-model averaging. Consistent with my earlier findings [4], the results indicate that the presence of large numbers of L2 speakers has no effect on complexity across languages when controlling for the estimated speaker population size.

## 2. Materials and Methods

Some material in this section is recycled from my prior publications [4,19,20] in accordance with the guidelines provided by the Text Recycling Research Project [23].

### 2.1. Original Data

*Population estimates and language information.* Basic information on different languages and genealogical classifications is taken from [24]. Information on geographical language areas is taken from [25]. Speaker population size and geographical range size estimates are taken from [26], who report the total number of L1 speakers based on information from *Ethnologue* [27] and calculate range sizes in km^2^ based on information from Global Mapping International [28]. Aggregated information on vehicularity and L2 proportions are taken from *Ethnologue* [27].

Languages with an EGIDS value of 0, 1, 2 or 3 are categorised as vehicular, while languages with an EGIDS value of 4 to 10 are categorised as non-vehicular. Aggregated *L2* speaker proportions are taken from [27] and from [29]. The different sources are merged via the three-letter language-specific ISO 639-3 code. For a critical yet balanced assessment of *Ethnologue’s* strengths and limitations, see Hammarström [9].

*Morphological complexity.* To construct an index of morphological complexity, ref. [30] extracted information on 28 relevant features of morphology from the *World Atlas of Languages Structures* [31] (WALS). For example, the WALS’s chapter 30A, “Number of Genders,” gives a range of 5 values from “None” to “Five or more”. These values are then mapped to the values 1 to 5, where higher values are indicative of higher complexity. The values of each feature are normalised to the interval [0,1]. The morphological complexity score *C* is then calculated by summing the normalised features divided by the number of available features. Let $f_{i}$ be the normalised value of feature *i*, and $N_{F}$ be the number of features that are available in the corresponding languages, and then *C* can be written as:

(1) $$C=\frac{1}{N_{F}}\times \sum_{i=1}^{N_{F}}f_{i}$$

Greater values are indicative of higher morphological complexity; for more details and a list of all used WALS features, cf. [30]. In total, there are 1713 languages with at least one available feature. It is important to note that the amount of available WALS information varies greatly for different languages [1,5], e.g., there are only 10 languages for which information on all 28 features is available [30], but there are 393 understudied languages with only 1 or 2 available features. To account for this data sparseness, separate analyses are conducted: (i) full: a full version that incorporates all languages that have information for at least one available WALS feature; and (ii) subset: a version for a subset of languages with available information for at least six features (50% of all languages have information on at least six features).

*Information-theoretic complexity.* The average per-symbol information content or entropy rate of a text can be interpreted as a measure of complexity [32,33]: the harder it is, on average, to predict upcoming text, the higher the entropy rate and the greater the complexity of the text as a whole [34,35,36,37]. I used estimates for the Gospel of Mark in more than 1000 different languages based on the Parallel Bible Corpus [24], which are taken from [38]. For languages with more than one available translation, entropy estimates are averaged.

Entropy rates are estimated on the basis of the non-parametric method of [13,36] that builds on the key idea of the Lempel–Ziv compression algorithm [39]. This method does not require any prior training, produces robust estimates without the need for very long strings as input and is able to take into account the very long-range correlations typical of literary texts [40,41] that are not captured by direct parametric Markovian or “plug-in” estimators [36]. If we represent a text t as a symbolic sequence of N characters, i.e., $t=\left\{c_{1},c_{2},\ldots ,c_{N-1},c_{N}\right\}$ where $c_{i}$ represents any character (including white spaces and punctuation marks) in the text at position *i*, the entropy rate can be estimated as [36]; cf. Equation (1):

(2) $$H_{t}=\;{\left[\frac{1}{N}\sum_{i=2}^{N}\frac{l_{i}}{{log}_{2}(i)}\right]}^{-1}$$

Here, the key quantity of interest is the match-length $l_{i}$. In order to determine the redundancy at position *i*, we examined the whole portion of the text up to (but not including) i and monitored how many of the initial characters of the text portion starting at *i* have already occurred in the same order somewhere in the preceding text, and recorded the length of the longest continuous substring. Our key quantity of interest $l_{i}$ was obtained by adding 1 to the longest match-length. More details of this approach can be found in [38].

In total, the dataset includes information for 2143 different languages, with 1088 data points for entropy rates and 1581 for morphological complexity. Of these languages, 1902 are categorised as non-vehicular, while the remaining 241 are vehicular. The median estimated speaker population size across languages is 15,000. These languages represent a total of 126 language families, with significant representation from families such as Niger-Congo (16.99%), Austronesian (14.61%), Trans-New Guinea (7.93%), Sino-Tibetan (5.23%), Afro-Asiatic (4.95%) and Indo-European (4.48%), among others. The data can be downloaded from https://dx.doi.org/10.6084/m9.figshare.c.4400675 (accessed on 8 November 2024).

### 2.2. Additional Data on Information-Theoretic Complexity

With respect to the information-theoretic complexity estimates used in my original paper [4], there are three potential issues: (i) complexity was estimated solely at the character level; (ii) complexity was calculated using a rather simple non-parametric statistical entropy estimation method; and (iii) language-specific estimates were based only on parallel translations of the Gospel of Mark of the Bible (see Section 2.1). To address these issues in what follows, I used information-theoretic complexity estimates derived from a multilingual database that I compiled [19,20].

Regarding (i), the estimation at the character level is problematic due to cross-linguistic differences in the mapping between phonemes and graphemes [17,18]. For example, languages with deep orthographies, like English, have inconsistent mappings (e.g., “ough” in “thought” vs. “through” vs. “dough”), while languages with shallow orthographies, like Spanish, have more consistent phoneme-to-grapheme correspondences (e.g., “a” in “casa” is always pronounced the same). To mitigate this problem, information-theoretic complexity in [20] is measured at multiple levels: characters, words and the supra-character but sub-word level, by applying byte-pair encoding (BPE) [42,43]. BPE is a sub-word segmentation technique that iteratively merges the most frequent pairs of characters or character sequences, creating sub-word units that capture meaningful linguistic patterns. It plays a crucial role in modern language modelling by effectively handling morphological variations and rare words, thereby enhancing model performance across diverse languages. Moreover, BPE’s ability to reveal language-specific sub-word patterns makes it particularly valuable in cross-linguistic investigations, as it highlights structural differences and typological features that are unique to each language, as recently discussed in [44].

Regarding (ii), the simplicity of the entropy estimator used in my initial paper [4], as described above, may have influenced the results. To address this, ref. [20] trained an ensemble of seven different types of language models on each document in the multilingual database, ranging from simple statistical n-gram models to state-of-the-art neural networks and transformer models. In this paper, I used the estimates from the best-performing language model for each document. For more details on language modelling and estimation, see [20].

Regarding (iii), the use of the Bible as a parallel text source poses significant challenges; for details, see [14,15,16]. Of particular importance is the fact that many Bible translations for minority languages, especially those produced by missionaries after World War II, were created with a specific missionary purpose in mind. These translations aim to convey the message in a manner that is easily understandable to readers or listeners, often including a considerable amount of redundant explicative content. This can complicate cross-linguistic analyses of the potential impact of social structure on language structure [16]. The compiled database helps to overcome this issue, as it includes 40 multilingual text collections encompassing a wide variety of parallel texts, such as religious texts, legal documents, movie subtitles and machine translations. Additionally, the database includes comparable corpora, which are not parallel but come from similar sources, such as newspaper articles, web crawls, Wikipedia entries, and system message translations from the Ubuntu operating system.

Overall, the database includes nearly 1.5 billion words across 3705 documents in 1104 languages. Of these languages, 882 are classified as non-vehicular, with the remaining 222 designated as vehicular. The median estimated speaker population size for these languages is 69,796. The dataset spans 99 language families, with Niger-Congo (19.38%), Austronesian (16.49%), Indo-European (9.06%), Trans-New Guinea (7.70%) and Otomanguean (4.35%) among the most represented. Further details on database compilation, including data pre-processing, document preparation, language modelling and complexity estimation methods, are available in [19,20]. The data can be downloaded from https://osf.io/xdwjc/ (accessed on 8 November 2024).

### 2.3. Additional Methods

To test if vehicularity significantly predicts information-theoretic complexity, I ran separate models with the entropy rate *h* as a measure of the information-theoretic complexity as the outcome on all three levels (words/characters/BPE). For *N* = 3705 individual documents, I fitted parametric linear mixed multi-level models (LMMs) of the form [45]:

(3) y=Xβ+Zu+ϵ

where **y** is the *N* × 1 vector of estimated values of *h*; **X** is the *N* × *p* design matrix of *p* covariates, including a *N* × 1 vector of ones for the intercept; β is the corresponding *p* × 1 vector of coefficients; ϵ is the *N* × 1 vector of residuals, **Z** is a matrix of random predictors; and **u** is a vector of random effects that are assumed to follow a normal distribution, with mean 0 and variance-covariance matrix **G**. The residual errors **ϵ** are assumed to follow a normal distribution, with mean 0 and variance matrix $\sigma^{2}I$; u⊥ϵ. As fixed effects, I considered fixed effects for vehicularity, the speaker population size and their interaction. In addition, the following control variables were considered: corpus type (parallel/comparable), text length in symbols and the number of countries in which each language is spoken.

All quantitative variables (including the outcomes) are log-transformed. Text length is measured in words for words as symbols and in characters on both the character and BPE levels. The rationale behind considering the number of countries as a potential covariate is to account for the varying degrees of pluricentrism [46]. For instance, languages such as English or Spanish are spoken in several countries and may, therefore, have different codified standard forms.

Random intercepts for the following groups were included: the writing script, corpus, macro-area, macro-family, country, sub-family and language. I crossed corpus, macro-area, macro-family and writing script, and explicitly nested countries within macro-areas and language within sub-family within macro-family. To account for variations within language families and geographical units [12,47], I included random slopes for population size, i.e., the effect of population size is allowed to vary across the following groups: macro-area, country, macro-family and sub-family. In models without a fixed effect for speaker population size, potential random slopes were also excluded since excluding the fixed effect for speaker population size while including random slopes would constrain $\beta_{x}$ to be zero and thus force the random slopes to be evenly distributed around a slope of zero.

Given the absence of clear theoretical or empirical reasons to determine which variables to include as controls, I adopted a multi-model inference approach [21] by sub-setting each full model, i.e., per symbolic level (words, characters, BPE), I generated a set of *R* = 17,600 candidate models with all possible variable subsets, which were then fitted to the data [20]. All models were fitted with gradient-based maximisation (maximal number of 20 iterations) and via maximum likelihood (ML). Per type and for each fixed effect, I then computed a frequentist model averaging (FMA) estimator over all *R* candidate models [21,48]:

(4) $${\tilde{\beta }}_{x}=\sum_{j=1}^{R}\omega_{j}\beta_{x,j}$$

where $\beta_{x,j}$ denotes the estimated fixed effect of variable *x* for model *j*, and $\omega_{j}$ is a weight computed as:

(5) $$\omega_{j}=\frac{e^{\left(-\frac{1}{2}\Delta_{j}\right)}}{\;\Omega }$$

where $\Omega =\sum_{r=1}^{R}e^{\left(-\frac{1}{2}\Delta_{r}\right)}$ represents the sum of weights for all *R* models. To compute $\Delta_{j}$, I used AIC, $\Delta_{j}={AIC}_{j}-{AIC}_{min}$, where ${AIC}_{j}$ denotes the AIC value computed for model *j* and ${AIC}_{min}$ represents the minimum AIC value over all *R* models. Note that in models where *x* does not appear, $\beta_{x,j}\equiv 0$. On this basis, I computed an FMA estimator of the standard error (SE) as [21]:

(6) $$SE({\tilde{\beta }}_{x})=\sum_{j=1}^{R}\omega_{j}\sqrt{{SE(\beta_{x,j})}^{2}+{\left(\beta_{x,j}-{\tilde{\beta }}_{x}\right)}^{2}}$$

where $SE(\beta_{x,j})$ denotes the estimated standard error of $\beta_{x,j}$ for model *j*. In models where *x* does not appear, I set $SE(\beta_{x,j})\equiv 0$. To assess the statistical significance, I computed a corresponding two-tailed *p*-value as $p=2\cdot \left(1-\Phi \left(\left|\frac{{\tilde{\beta }}_{x}}{SE({\tilde{\beta }}_{x})}\right|\right)\right)$, where Φ() denotes the cumulative standard normal distribution. Since the Akaike weights $\omega_{j}$ can be viewed as approximate probabilities, indicating the likelihood of each model being the best one given the data, $\omega_{j}$ can be used to estimate the relative importance of variable *x*, computed as [21]:

(7) $$\sigma_{x}=\sum_{j=1}^{R}\omega_{j}c_{x,j}$$

where $c_{x,j}$ is a binary indicator that is equal to 1 if *x* is explicitly in model *j* and 0 otherwise [21]. The larger $\sigma_{x}$, the more important *x*. I computed $\sigma_{x}$ for both the fixed effects and the random effects/slopes.

While the LMM multi-model structure specified above offers a potential solution that better accounts for both model uncertainty and the genealogical and geographic relatedness of languages compared to the approach applied in my original paper, this approach may still be incomplete if there are patterns of relatedness within random groups for which no random slopes are included—such as those below the level of sub-families or countries [12]. For this reason, an approach where relatedness is explicitly modelled as a function of spatial or phylogenetic distance is potentially preferable [12]. For the analysis of information-theoretic complexity, however, such an approach is neither feasible nor desirable due to the unbalanced nature of my multilingual database (for details, see [19]). In this context, an LMM-based method has the significant advantage of providing a principled way to account for variations between group levels while simultaneously avoiding overfitting within groups [49]. On the other hand, the data available for morphological complexity are balanced in this respect, making it possible to estimate a spatial autoregressive (SAR) model, which can be written as [22]:

(8) $$y=X\beta +{\left(I-\varphi W\right)}^{-1}\epsilon$$

where, in addition to the above (Equation (3)), φ is the autoregressive parameter; W is an *N* × *N* spatial weight matrix, representing the spatial structure between observations; and **I** represents the identity matrix. I constructed two different inverse distance matrices, (i) to control for geographic proximity, a matrix $W_{geo}$ based on the geographical distances between languages was constructed, and (ii) to control for phylogenetic relatedness, a matrix $W_{phylo}$ based on a phylogenetic similarity matrix provided by [50] was used. In both cases, the matrix elements are equal to the reciprocal of distance that are then normalised using spectral normalisation. In total, information on both morphological complexity, $W_{geo}$ and $W_{phylo}$, is available for *N* = 1443 languages. As above and in my original paper, I present separate analyses for the full and subset versions.

As covariates, I considered vehicularity and three quantitative variables: (i) language speaker population size, (ii) language range size and (iii) number of countries. Additionally, I included separate first-order interactions between vehicularity and each quantitative variable. Once again, I adopted an FMA inference approach by sub-setting each full model. For both the full dataset (*N* = 1443) and the subset (*N* = 804), I generated a model space of *R* = 35 SAR models with all possible variable subsets, fitting them to the data while modelling the spatial dependence based on either $W_{geo}$ or $W_{phylo}$. I then proceeded, as described above, to compute averaged estimates, statistical significance and the relative importance of each considered variable. For the FMA approach, I used an estimator that maximises the likelihood $\hat{L}$. Additional analyses were carried out using a generalised spatial two-stage least-squares estimator (GS2SLS) [51] that allows for the inclusion of more than one spatially lagged error term but does not maximise $\hat{L}$.

One of the advantages of an FMA-based approach is its ability to explore large model spaces comprehensively. In this context, note that, as written above, vehicular languages are defined as those with an EGIDS value of 0, 1, 2 or 3 (see Table 1). According to *Ethnologue’s* classification system, all of these EGIDS categories are expected to have a significant number of L2 speakers, with a plausible assumption that the lower the value, the higher the L2 proportion.

This thus provides the possibility of a more in-depth test of the linguistic niche hypothesis. To achieve this, I constructed a second model space consisting of *R* = 8695 candidate models, where I considered binary indicators for ‘0—International’, ‘1—National’, ‘2—Regional’ and ‘3—Trade’. As before, I included three quantitative variables—(i) language speaker population size, (ii) language range size, and (iii) number of countries—along with first-order interactions between the quantitative variables and the binary EGIDS indicators.

All statistical analyses were carried out using Stata 18/MP. Commented code to reproduce all results is available at https://osf.io/fypx5/.

## 3. Results

### 3.1. A Rebuttal of the Critique of Kauhanen, Einhaus and Walkden (2023)

**Figure 1:**
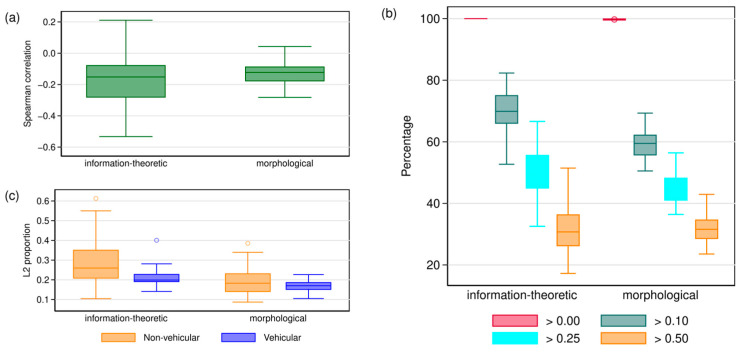
Descriptive results of KEW’s multiple imputation approach. Per type of complexity (information-theoretic or morphological), computations are based on the 100 completed samples from KEW’s multiple imputation analysis. (**a**) Spearman correlation between the imputed L2 proportion and speaker population size and (**b**) percentage of languages that have an L2 proportion of (i) more than 0, (ii) more than 0.10, (iii) more than 0.25 and (iv) more than 0.50 per type of complexity (information-theoretic or morphological). (**c**) Median L2 proportion for non-vehicular and vehicular languages per type of complexity.

KEW criticised the use of vehicularity as a proxy of whether a language is likely to have significant numbers of L2 speakers. They stated (p. 3): “In Koplenig’s analysis, languages with an EGIDS score of 3 or lower are defined to be vehicular, the rest being non-vehicular”. I believe it is important to point out that this is not my definition or mapping but how languages are categorised by the *Ethnologue* [10]. KEW (p. 3) rightfully pointed out that a “considerable number of non-vehicular languages are reported by Ethnologue to be used as an L2 even though no numerical estimate of L2 users is given.” I fully agree that this inconsistency is problematic and that it is thus important to ask if vehicularity is a good proxy for whether a language is used as an L2. I explicitly discussed this in the concluding section of my original paper. Here, I quote the editors of the *Ethnologue*: “Based on the use of the phrase ‘vehicular language’ by some as a synonym for lingua franca, we use the term vehicular to refer to the extent to which a language is used to facilitate communication among those who speak different first languages. If a language is characterised here as being Vehicular, it is used by others as an L2 in addition to being used by the community of L1 speakers.” ([10]; see also Figure 1 therein). Based on this assessment, I believe that it is appropriate to use vehicularity in order to test the linguistic niche hypothesis: a language that is defined as vehicular should—according to the *Ethnologue*—be a language that is “used for communication between strangers” (KEW, p. 1; also see [52]) and “should have a significant number of L2 users” [7], p. 20. Thus, if the linguistic niche hypothesis holds, we should expect that there is a statistical association between vehicularity and complexity. In my paper [4], I demonstrated that this is not the case for either morphological or information-theoretic complexity when controlling for speaker population size.

Importantly, the problem of there being non-vehicular languages for which *Ethnologue* reports a proportion of L2 users greater than 0 is not concealed by me, but explicitly mentioned in Section 2.2 of my paper [4] and—as also mentioned therein—additional analyses are presented and discussed in Section 7 of the accompanying supplementary material, where languages categorised as non-vehicular but with L2 proportions greater than zero are excluded. The reported results generally support the results presented in the main part of the paper. KEW do not mention or take these analyses into account.

In a set of further statistical analyses, I used vehicularity to impute missing values: In correspondence with the categorisation scheme of the *Ethnologue* [7], non-vehicular languages with no available information on L2 users are assigned an L2 proportion of 0. KEW are right to point out that this step is worth discussing since this zero-imputation strategy affects almost all non-vehicular languages. Importantly, however, imputed values are only used for the non-parametric Spearman correlation analyses. Here, I tested whether there is a significant (determined by non-parametric permutation tests) monotonic relationship between (morphological or information-theoretic) complexity and the L2 proportion after removing the effect of speaker population size and vice versa (correlating complexity and speaker population size while controlling for the L2 proportion). Since, as mentioned in the paper [4] (Section 2.6), Spearman correlation coefficients and part Spearman correlation coefficients can be computed as a Pearson’s correlation coefficient on the ranks of the two variables, where the zero-imputation strategy implies that all non-vehicular languages are assigned the lowest rank in each analysis—an assumption that I believe is reasonable but worthy of discussion. KEW (Section 5.1) presented three so-called complete case analyses, where all cases with missing information are removed (no imputation). Using parametric LMMs (morphological or information-theoretic) complexity is predicted by the fixed effects of the (log of) population size and the L2 proportion and a random intercept for the language family. Additionally, the two models with morphological complexity as the outcome also include a random intercept for the linguistic area. Based on their results (Table 2 and Table 3), KEW argued that “population size and the proportion of L2 speakers have a declining effect on morphological complexity, and both predictors are statistically significant.” (p. 6). For information-theoretic complexity as the outcome (Table 4), KEW found no evidence “for an effect of either the proportion of L2 speakers or population size” (p. 6). There are two major methodological problems with KEW’s models: (i) KEW did not include any random slopes in their models due to convergence issues with the software package they used to fit LMMs; and (ii) the estimates in KEW’s models are derived by restricted maximum likelihood (REML). This is highly problematic because they use Akaike’s information criterion for model selection, which is defined as $AIC=-2log(\hat{L})+2k$, where lower values indicate a better model and where $log(\hat{L})$ represents the log-likelihood of the model, and *k* represents the number of estimated parameters [21]. For example, on p. 6, KEW argued that they “do not include interactions between the covariates in any of our models, as doing so always leads to a worse model when quantified on AIC”. However, when different sets of fixed effects are considered, estimates must not be derived by REML, but by ML [53,54,55] since REML does not provide the full likelihood $\hat{L}$ of the model, using AIC with REML is inappropriate (REML only provides a restricted likelihood that accounts for random effects but not for thefixed effects). To solve both (i) and (ii), I generated a set of 72 candidate models consisting of all possible combinations of fixed effects for speaker population size, the L2 proportion and their interaction, as well as crossed random intercepts for language family and linguistic area and random slopes for both speaker population size and the L2 proportion for both random effects (for 16 out of 171 languages, information on the linguistic area were missing. In these cases, I manually imputed the data using the original definitions provided by the Autotyp project [25]. Further noting that here and in what follows, models that include interactions are only considered if the corresponding main effects are also included in the model). I used Stata/MP 18 for the LLMs, estimates were derived by ML, and models were fitted with gradient-based maximisation. Of a total of 216 models, 214 (or 99.07%) converged to an optimal solution, thus pointing towards problems in KEW’s analyses. Table 2 summarises the results. I first checked for all three outcome versions of whether the model with the lowest AIC includes the L2 proportion as a fixed effect; row 2 of Table 2 shows that this is only the case for the versions where morphological complexity is the outcome. This means that including the speaker L2 proportion does not improve the model fit in the case of information-theoretic complexity. I then extracted the best models that include a fixed effect for the L2 proportion. Rows 4—6 list the model structure per outcome. Row 6 shows that there is only evidence for a significant effect of the L2 proportion for morphological complexity as the outcome for the subset version, i.e., when only languages with available information for at least six WALS features are considered, while for both other outcome versions, there is no evidence for a significant effect of the L2 proportion on complexity at any standard level of significance. The results obtained are not altered when an alternative version of Aikaike’s information criterion, AICc [56], is used instead of AIC. AICc accounts for the sample size by including an additional bias correction term; see also Burnham and Anderson [21].

However, as pointed out in my original paper [4] and discussed further in Section 2 of the supplementary material for a similar sample, it is not clear whether any of the three samples are unbiased: (i) row 8 of Table 2 shows that almost all languages in all three samples have an L2 proportion that is greater than zero with a median estimate (row 9) of more than 15%, which seems rather high given the assumption of the linguistic niche hypothesis that most languages have almost no L2 speakers.

(ii) Compared to the median estimated speaker population size of 7000 for all languages listed by the *Ethnologue* [1], the median estimated speaker population size in the samples ranges between 786,500 and 2,942,020 (row 10). To place this into perspective, I drew 1,000,000 random samples of all 6880 living languages listed by *Ethnologue* [7], with each sample consisting of 100 languages. For each sample, I computed the median population size. The median estimate for the median population size is 8000, and the sample with the highest median population size is 51,000. Thus, the three samples used for KEW’s complete case analyses have a median population size that is several orders of magnitude larger than what we would expect from a random sample, and the probability of randomly drawing a sample like the one used in the complete case analyses above is less than one in a million. This calls into question the appropriateness of standard parametric frequentist approaches [16,57], which is why, in my original paper [4], I used non-parametric tests that do not make any assumptions regarding the stochastic mechanism that generated the data [58].

(iii) There is no positive Spearman correlation between the estimated size of the speaker population and the estimated proportion of L2 speakers in any of the three samples (row 11). As I have written above and in my original paper [4], this is actually a key assumption of the linguistic niche hypothesis

Against this background, I would like to reiterate that KEW are right to point out that the imputation step is worth discussing; I do so myself in Section 2.2 of my original paper [4] and present complete case analyses without any imputation (similar to that presented by KEW, but without making parametric assumptions) in Section 10 of the supplementary material of my original paper [4]. Again, KEW neither mention nor seem to take this into account.

In their second set of analyses, KEW utilised a technique called multiple imputation to fill in the missing data [59,60,61]. As I will show in what follows, while the idea of imputing data in a scenario with a missingness rate of approximately 92% (KEW, p. 10) may seem highly desirable at first glance, a closer examination reveals that KEW’s approach suffers from severe and systematic biases that render it an unreliable method for accurately filling in missing data in this context. Multiple imputation offers a flexible, simulation-based technique to handle missing data consisting of three steps [62]: (i) setting up an imputation model and generating *m* imputations (completed datasets), in KEW’s analyses, *m* = 100; (ii) the completed datasets are separately analysed with standard statistical techniques; (iii) the results obtained in (ii) are pooled to provide estimates of the parameters of interest, accounting for the uncertainty due to missing data. KEW rightfully pointed out that the technique is based on the assumption that the data are either missing completely at random (MCAR) (i.e., missingness for a variable *x* is unrelated to the observed values of both other variables and the unobserved values of *x*), or missing at random (MAR) (i.e., missingness on *x* is uncorrelated with the unobserved value of *x* after other variables in the dataset have been used to predict missingness on *x)*. Or, explained differently, after controlling for the observed variables, the probability of missingness is independent of the true value of *x.* If this is not true, the data are said to be missing not at random (MNAR), if the value of *x* itself predicts missingness [63,64]. In general, multiple imputation methods assume that data are MCAR or MAR and not MNAR. KEW (p. 11) argued that “In the current state of understanding, we feel it would be premature to conclude one way or the other, but we point out that any argument to the effect that L2 speaker proportions are MNAR would need to specify a mechanism whereby such missingness arises.” Unfortunately, there is no formal test to answer this question since, as written above, the data that would be needed to determine this are, themselves, missing. However, there is a different way of representing MAR [65]: MAR implies that the distribution of *x*, given our imputation model, is the same whether or not *x* is observed. If the data are MNAR, however, the chance of observing a value of *x* depends on *x*, even after conditioning on our model. In this case, based on our imputation model, the observed data do not provide information on how the missing values differ from the observed ones [65]. As an imputation model (ℳ_KEW_) to impute L2 proportions, KEW used an LMM with the (logit-transformed) L2 proportion as outcome, random intercepts for (either morphological or information-theoretic) complexity, the log of population size and the log of language range size. Now, the question is whether the chance of observing a value of the L2 proportion really does not depend on this value itself after conditioning on ℳ_KEW_. I would argue that it does, or do we really believe that ℳ_KEW_ tells us *all* there is to know regarding the question of how the missing values of the L2 proportions are different from the corresponding observed values? KEW (p. 11) sketched one such mechanism: a “greater proportion of L2 speakers in a speech community increases, in general, the access that outsiders have to that community, and hence also increases the likelihood of the demographic variable of L2 speaker proportion being recorded by field typologists.” This seems like a reasonable assumption: the smaller the value of the L2 proportion, the bigger the chance that this value is missing. I would even say that missing the L2 proportion could be used as a standard example of an MNAR type since we almost exclusively observe higher values of L2, as written by KEW (p. 3); only in four cases does the *Ethnologue* provide “an actual numerical zero proportion estimate.” But, since there is no test to formally determine this, different researchers can have different opinions regarding the type of missingness. Nevertheless, we can test the efficiency of ℳ_KEW_ in order to find out if the multiple imputation will produce unbiased estimates, even in the presence of large proportions of missing data. KEW (p. 11) cited Madley-Dowd et al. [66], who demonstrated that missingness up to “90% is tolerated by the method as long as the imputation model includes all necessary predictors”. Madley-Dowd et al. [66] showed that this strongly depends on the strength of the imputation model. In their simulation study, strength is determined using the coefficient of determination *R*^2^, which measures the proportion of variance in the outcome that is predictable by the imputation model. To provide unbiased estimates in the presence of high rates of missingness, Madley-Dowd et al. [66] showed that the imputation model needs to be almost perfect with an *R*^2^ as high as 92%. To test this for ℳ_KEW_, I re-ran the specified models and computed an *R*^2^ of ~44% for morphological complexity as a covariate in ℳ_KEW_ and corresponding *R*^2^ of ~32% for information-theoretic complexity as a covariate in ℳ_KEW_.

For such an LMM, I computed the so-called conditional *R*^2^, i.e., the variance explained by both the fixed effects and the random intercept [67]. Note that in the fully observed data, there is information for morphological complexity as a covariate in the imputation model for only 28 language families. However, as KEW wrote (p. 8), their model imputes information for 122 families. This means that ~77% of all language families are systematically missing. Similar quantities are obtained for information-theoretic complexity, where information is systematically missing for ~84% of all language families. To place this into perspective, I computed an LMM with the log of population size as the outcome and a random intercept for language family for all available data points (*N* = 2143). The variance of the random intercept is 6.63. I then re-ran the model but restricted the computation to data points where the L2 proportion is non-missing (*N* = 171). Here, the variance of the random intercept is about 3.5 times higher, with a value of 24.10. Jolani [68], who developed the imputation method used by KEW, discusses potential biases in the estimated random effects parameters that can arise from systematic missingness. Jolani presented simulation results for systematically missing rates of up to 30%; it does not seem unlikely that problems could be more pronounced for missingness rates of more than 75%.

If we compare KEW’s results with the results of Madley-Dowd et al. (Table 2 in [66]), we find out that with a missingness rate of 90%, the reduction in standard error (compared to a complete cases analysis model) is arguably very unimpressive, ranging between ~0% and ~9%, to be exact for *R*^2^ = 52%, the error reduction is 8.86%, for *R*^2^ = 40%, the error reduction is 2.18% and for *R*^2^ = 36%, the error reduction is 0.11%. As a guide to test for efficiency gains, Madley-Dowd et al. [66] showed that the fraction of missing information (FMI) is a valuable quantity (ranging between 0 and 1) for determining the potential efficiency gains from multiple imputation: The FMI is a measure specific to each parameter that quantifies the information loss caused by missing data, while also considering the amount of information preserved by other variables in the dataset [66]. Its interpretation is similar to an *R*^2^, so an FMI of, say, 0.2 means that 20% of the total sampling variance can be attributed to missing data. A high value indicates a problematic variable [64]. KEW reported FMI values for both their imputations models but did not interpret them: for information-theoretic complexity as a outcome (Table 6 of KEW and so on), the FMI value is ~62%, and for morphological complexity as a outcome (Table 5), the FMI value is ~89%. This alone shows that ℳ_KEW_ does not provide much information about the missing values, especially in the case of morphological complexity, where KEW reported a negative significant effect of the L2 proportion on complexity. For information-theoretic complexity, there is no indication of an effect at any standard level of statistical significance. To further investigate this, I extracted all 100 imputed completed samples based on ℳ_KEW_ from the code provided by KEW. First, I computed the Spearman correlation between the imputed L2 proportion and speaker population size for each completed dataset and for both types of complexity. Plot (a) of Figure 1 presents the results: for both types of complexity, the Spearman correlation is negative in most samples, and in 75% of all samples, the Spearman correlation is lower than −0.08/−0.09 for information-theoretic/morphological complexity. This seems rather implausible and—as written above and in my original paper [4]—contradicts a basic assumption of the linguistic niche hypothesis.

For each sample, I then computed the percentage of languages that have an L2 proportion of (i) more than 0, (ii) more than 0.10, (iii) more than 0.25 and (iv) more than 0.50. Plot (b) of Figure 1 visualises the results. For information-theoretic complexity, all languages have an L2 proportion > 0; for morphological complexity, the median across the samples is 99.74%. Both results do not seem plausible with respect to the linguistic niche hypothesis. For the remaining quantities, the results seem to be equally implausible: for information-theoretic complexity, the median percentages are 69.89% for the L2 proportion > 0.10, 49.44% for the L2 proportion > 0.25 and 30.74% for the L2 proportion > 0.50; for morphological complexity, the median percentages are 59.48% for the L2 proportion > 0.10, 44.47% for the L2 proportion > 0.25 and 31.57% for the L2 proportion > 0.50. In my view, it is very hard to argue that a model that assumes that almost a third of all languages have an L2 proportion of over 50% reflects the linguistic reality.

Finally, I computed the median estimated L2 proportion in each sample for both non-vehicular, i.e., ℒ_non-vehic_, and vehicular languages, i.e., ℒ_vehic_. Plot (c) of Figure 1 presents the results: for both types of complexity, the completed datasets based on ℳ_KEW_ show a lower L2 proportion for the vehicular languages compared to non-vehicular languages in the majority of cases (80 out of 100 for information-theoretic complexity and 68 out of 100 for morphological complexity). Specifically, the median ℒ_vehic_ is 0.20 for information-theoretic complexity and 0.17 for morphological complexity, whereas the median ℒ_non-vehic_ is 0.26 for information-theoretic complexity and 0.18 for morphological complexity. As written above and in my original paper [4], this completely contradicts the categorisation scheme of the *Ethnologue* and basic typological intuitions.

### 3.2. Updated Results

Table 3 summarises the results for information-theoretic complexity as the outcome. Consistent with the findings from my original paper [4], while there is a stable positive effect of population size on information-theoretic complexity, no evidence is found for an effect of vehicularity at any of the three symbolic levels (words, characters and BPE). Additionally, the $\sigma_{x}$-values (Equation (7)) reflecting relative variable importance indicate that vehicularity is not an important factor in explaining information-theoretic complexity (all $\sigma_{x}$-values < 0.70). In contrast, all $\sigma_{x}$-values for speaker population reach the maximum value of one, underscoring its importance in predicting information-theoretic complexity. This suggests that my initial results generalise across different language models, symbolic levels and text types.

Table 4 summarises the results for the SAR model, averaging with *R* = 35 candidate models, applied to both (i) the full dataset and the subset that includes only languages with at least six available WALS features and (ii) two different weight matrices, $W_{geo}$ and $W_{phylo}$. Consistent with my original paper [4], there is strong evidence of a statistically significant negative effect of population size on morphological complexity (at *p* < 0.001) in all four investigated scenarios. Three out of four corresponding $\sigma_{x}$-values are maximal, with the remaining value also indicating high variable importance ($\sigma_{x}$ = 0.97). The only other variable that reaches statistical significance is language range size, which shows a significant effect on morphological complexity in three out of four cases. Interestingly, however, all three corresponding ${\tilde{\beta }}_{x}$-values are positive. Based on the linguistic niche hypothesis, one would expect the opposite [1].

Regarding vehicularity, the ${\tilde{\beta }}_{x}$-value is negative in all four scenarios, which, at first glance, aligns with the linguistic niche hypothesis. However, in none of these cases is the corresponding coefficient significantly different from zero (all *p*-values > 0.05). Interestingly, all corresponding $\sigma_{x}$-values indicate high importance for vehicularity.

To understand this seemingly counter-intuitive result, I computed the $\sigma_{x}$-value of all *R* = 13 candidate models that do not include population size as a covariate. In all four scenarios, the resulting $\sigma_{x}$-value is very low, with three out of four values below 0.001 and the remaining value below 0.05. This suggests that the apparent importance of vehicularity is actually driven by the strong effect of population size. To further explore this, I ran a SAR model of morphological complexity using population size as the predictor, with two spatially lagged error terms specified by each weighting matrix, using the GS2SLS estimator. From this model, I calculated uncorrelated residuals, which serve as estimates of the uncorrelated error term. I then computed the Pearson correlation between these residuals and vehicularity. For both the full and the subset versions, the resulting negative correlation coefficient was insignificant (*p* = 0.071 for the full and *p* = 0.338 for the subset).

Next, I reversed the analysis: I ran the SAR model with morphological complexity as the outcome and vehicularity as the predictor, including the two error terms. Again, I calculated uncorrelated residuals and correlated them with population size. In this case, for both the full and the subset versions, the negative correlation coefficients were significant (*p* < 0.01 for the full version and *p* < 0.05 for the subset version). This further indicates that the observed effect of vehicularity is actually driven by differences in population size.

Table 5 presents the findings from the larger candidate space (*R* = 8695). The results from this FMA align well with those based on the smaller model space (Table 4): the only consistent evidence for any effect on morphological complexity is the negative ${\tilde{\beta }}_{x}$-value of population size across all four scenarios (all *p*-values < 0.001). Similarly, language range size shows a significant positive effect on morphological complexity in three out of four cases, with the exception being for the full version when $W_{phylo}$ is the autoregressive structure. With respect to the four binary EGIDS indicators, Table 5 shows that there is statistically significant evidence for an effect only in one out of sixteen cases: for the subset version with $W_{phylo}$ as the weight matrix, the ${\tilde{\beta }}_{x}$-value of −0.5257 for the binary indicator for ‘3—Trade’ is significant at *p* < 0.05. All other ${\tilde{\beta }}_{x}$-values do not reach statistical significance. In addition, none of the first-order interactions passes the significance test. The fact that we find almost no significant negative effects thus constitutes strong evidence against the linguistic niche hypothesis.

Regarding relative variable importance, only the $\sigma_{x}$-values for population size are consistently above 0.90. For the EGIDS indicator variables, only five $\sigma_{x}$-values are above 0.90. As for the small candidate space, I computed the $\sigma_{x}$-value of all *R* = 803 candidate models that do not include population size as a covariate. In all four scenarios, the resulting $\sigma_{x}$-value is below 0.001. Again, this indicates that the results are driven by the strong effect of population size.

## 4. Discussion

In sum, I would like to thank KEW for providing me the opportunity to revisit the relationship between language complexity and the proportion of non-native speakers. However, as pointed out above, I had already addressed their two main points of critique in my original paper [4]. In this context, I would like to thank one of the reviewers of my original paper, to whom I owe the consideration of these two points of criticism—during peer review, the reviewer pointed out that both “the relationship between the two variables and the fact that not all languages with a vehicularity of 0 have 0 L2 speakers needs to be dealt with openly” and that there needs to be an analysis that “removes the 78 languages with a vehicularity index of 0 and a proportion of L2 speakers > 0”. Here, I refer the interested reader to the review reports, which I have deliberately chosen to make freely available online.

While KEW acknowledged and apologised for their oversight in personal communication, they did not retract their paper or issue any form of amendment or clarification. I, therefore, used the first part of the Results section (Section 3.1) to address their criticism. I hope to have convincingly demonstrated that the alternative analyses offered by KEW do not stand up to closer scrutiny: (i) Only one in three linear mixed-effects model analyses based on complete cases supports the linguistic niche hypothesis at all, and there are good reasons to doubt the appropriateness of the samples used to test for an effect of L2 proportions on complexity. (ii) The multiple imputation analyses suffer from similar biases, and it is clear from the interpretation of the FMI values reported by KEW that the imputation model does not provide much information about the missing values.

Nevertheless, KEW and I might agree that neither non-imputation nor imputation of L2 proportions is an ideal strategy. This brings us back to the use of vehicularity as an indicator of high L2 vs. low L2 languages. To drive home my point, let me provide an illustrative example that shows why it is possible to use such proxy variables to test claims between continuous variables: In a study examining the relationship between occupational exposure to a certain chemical and the risk of developing a specific health outcome, researchers may want to accurately measure the level of exposure to the chemical for each participant. However, it may be challenging to obtain accurate measurements of exposure, especially if the exposure occurred in the past or if the exposure was intermittent. Instead, they may rely on the assessment of medical experts, such as occupational health physicians, to classify each participant as having either high or low exposure based on their job history, work practices and other relevant factors. While this strategy does not seem to be perfect, and information certainly becomes lost when turning a continuous variable into a categorical one, it seems justified to statistically compare the incidence of the specific health outcome between the high- and low-exposure groups to assess whether there is a significant association between exposure to the chemical and the risk of developing the health outcome.

In our scenario, exposure to the chemical element is the proportion of L2 speakers, the outcome is language complexity, and the occupational health physicians are the field linguists of the *Ethnologue* that classify languages into high L2 language, i.e., vehicular languages, and for low L2 language, i.e., non-vehicular languages. Based on this logic, I aimed to demonstrate in the second part of the Results section (Section 3.2), using additional data on information-theoretic complexity and quantitative methods that better account for phylogenetic relatedness and geographic proximity, that when comparing a high L2 language with a low L2 language, both are statistically indistinguishable in terms of their morphological or information-theoretic complexity, provided that both languages have a comparable speaker population size. Furthermore, I conducted a more granular analysis using binary indicators for several EGIDS values that, according to the *Ethnologue*, should correspond to a substantial number of L2 speakers. This analysis also fails to support the linguistic niche hypothesis.

In the Appendix, I present the results of two additional analysis approaches, both based on a semi-parametric spatial filtering technique, which is outlined in Appendix A. The focus is on two key points:

(i) This paper primarily uses FMA, which is a method that integrates information from multiple plausible models while accounting for resulting uncertainty in the estimation process [21,48]. However, there are influential critiques cautioning against its naïve application. In particular, critics make the case against using model-averaged coefficients based on AIC weights [69,70,71,72,73]. While [21] claimed that AIC weights can be interpreted as model probabilities, those weights are only approximate, and as such, it can be problematic to interpret them as estimates of the predictor variable’s importance [70,71]. As an alternative to FMA, I thus considered Bayesian model averaging in Appendix B, as it provides a principled and unified way to estimate the model weights as posterior model probabilities that are readily interpretable [74,75].

(ii) Statistical significance was assessed using parametric frequentist approaches, whose appropriateness I called into question in my original paper [4]. In Appendix C, I present the results of a non-parametric test that does not make any assumptions regarding the stochastic mechanism that generated the data [58].

For results, see Table A1, Table A2, Table A3 and Table A4.

The results of both approaches are closely aligned with the findings presented in the main paper.

In a recent paper, Shcherbakova et al. [76] followed my suggestion to use vehicularity as an indicator of the proportion of L2 speakers. Using Grambank, a novel and extensive database of grammatical features [77], and employing statistical methods that differ from mine to account for the effects of genealogical and geographic non-independence of languages [78], they found that the only effects of vehicularity on grammatical complexity were weakly or moderately positive. Consistent with the results presented in both my original paper [4] and this paper, their findings provide no support for the “specific claim of the ‘linguistic niche hypothesis’ that grammatical complexity should reduce with an increased number of non-native speakers” [76]. The title of their paper neatly sums this up nicely: “Societies of strangers do not speak grammatically simpler languages”.

Now, critiques like KEW could argue that the categorisation of languages into high L2/vehicular and low L2/non-vehicular by the *Ethnologue* is incorrect. However, then this begs the question: If we do not trust the *Ethnologue* regarding this categorisation, why should we trust them regarding the—arguably more challenging—assessment of both the number of L1 and L2 speakers? As the saying goes—you cannot have your cake and eat it.

All in all, I thus do not have the impression that KEW’s critique weakens the argumentation laid out in my original paper in any way. Moreover, the updated data and analyses provided here indicate that there is still no evidence for an effect of the proportion of non-native speakers on language complexity.

## Figures and Tables

**Table 1:** Ethnologue’s EGIDS scale: column 1: EGIDS value and label; column 2: type (vehicular/non-vehicular); column 3: description taken from [10]; and column 4: three example languages taken from my dataset.

EGIDS Value	Type	Description	Examples
0—International	vehicular	“The language is used internationally for a broad range of functions.”	English, Mandarin Chinese, Spanish
1—National		“The language is used in education, work, mass media, government at the nationwide level.”	Bengali, Standard German, Ukrainian
2—Regional		“The language is used for local and regional mass media and governmental services.”	Gujarati, Igbo, Uyghur
3—Trade		“The language is used for local and regional work by both insiders and outsiders.”	Hakha Chin, Tagalog, Yue Chinese
4—Educational	non-vehicular	“Literacy in the language is being transmitted through a system of public education.”	Bhojpuri, Javanese, Santhali
5—Written		“The language is used orally by all generations and is effectively used in written form in parts of the community.”	Hakka Chinese, Madura, Sunda
6a—Vigorous		“The language is used orally by all generations and is being learned by children as their first language.”	Central Pashto, Central Atlas Tamazight, Sanʽani Arabic
6b—Threatened		“The language is used orally by all generations but only some of the child-bearing generation are transmitting it to their children.”	Aceh, Occitan, Peripheral Mongolian
7—Shifting		“The child-bearing generation knows the language well enough to use it among themselves but none are transmitting it to their children.”	Breton, Central Lalo, Tu
8a—Moribund		“The only remaining active speakers of the language are members of the grandparent generation.”	Baniwa, Soqotri, Yaqui
8b—Nearly Extinct		“The only remaining speakers of the language are members of the grandparent generation or older who have little opportunity to use the language.”	Qimant, Ratahan, Soo
9—Dormant		“The language serves as a reminder of heritage identity for an ethnic community. No one has more than symbolic proficiency.”	Clallam, Mogholi, Quileute
10—Extinct		“No one retains a sense of ethnic identity associated with the language, even for symbolic purposes.”	Madngele, Warrgamay *

* Note that for EGIDS value = 10, there are only two available languages in the used data.

**Table 2:** Overview of the results of the complete case linear mixed multi-level model analyses for each outcome. *β*_logPop_—estimated coefficient for the log of speaker population size; *β*_L2prop_—estimated coefficient for the L2 proportion; *β*_interaction_—estimated coefficient for the interaction between the L2 proportion and the log of speaker population size.

Row		Outcome		
		MC		H
1		Version: Full	Version: Subset	
2	Does the best model include a fixed effect for the L2 proportion?	Yes	Yes	No
3	Fixed effects	Population, L2 proportion and their interaction	Population and L2 proportion	Population and L2 proportion
4	Random intercepts	Family and Area	Area	Area
5	Random slopes	L2 proportion for Family	-	-
6	Estimated effects for the best model that includes a fixed effect for the L2 proportion, shown are the estimated beta coefficients for each fixed effect (parametric *p*-values in parentheses)	*β*_logPop_ = −0.010 (*p* = 0.138) *β*_L2prop_ = −0.065 (*p* = 0.738) *β*_interaction_ = −0.026 (*p* = 0.134)	*β*_logPop_ = −0.013 (*p* < 0.01) *β*_L2prop_ = −0.217 (*p* < 0.01)	*β*_logPop_ = 0.032 (*p* < 0.01) *β*_L2prop_ = −0.117 (*p* = 0.212)
7	Number of languages in the sample	148	101	94
8	How many languages in the sample have an L2 proportion > 0?	97.30%	96.04%	100.00%
9	Median L2 proportion	0.16	0.16	0.19
10	Median speaker population size	786,500	1,480,000	2,942,020
11	Spearman correlation between speaker population size and the L2 proportion (non-parametric permutation *p*-value in parentheses)	−0.074 (*p* = 0.352)	−0.025 (*p* = 0.352)	−0.151 (*p* = 0.140)

**Table 3:** Linear mixed multi-level frequentist model averaging results for information-theoretic complexity as the outcome computed on three different symbolic levels (words/characters/BPE). Per symbolic level, quantities are computed based on *R* = 17,600 candidate models. $\sigma_{x}$-values (Equation (7), rounded to two decimal places) reflect the relative importance of each variable (both fixed and random), with higher values indicating a more important variable. Values above 0.90 are shown in bold.$\;{\tilde{\beta }}_{x}$-values (Equation (4), rounded to four decimal places) are computed for the fixed effects only. ${\tilde{\beta }}_{x}$-values that pass a two-tailed parametric significance test at *p* < 0.05 are shown in bold. Sig. *** (**) [*] indicates that *p* < 0.001 (*p* < 0.01) [*p* < 0.05].

Type	Variable	Words		Characters		BPE	
		$\sigma_{x}$	${\tilde{\beta }}_{x}$	$\sigma_{x}$	${\tilde{\beta }}_{x}$	$\sigma_{x}$	${\tilde{\beta }}_{x}$
**Fixed effects**	Vehicularity	0.39	−0.009	0.66	−0.001	0.69	−0.003
	Population size	**1.00**	0.009 ***	**1.00**	0.011 ***	**1.00**	0.015 ***
	Population size x Vehicularity	0.16	0.001	0.22	−0.000	0.23	−0.001
	No. of countries	0.36	−0.002	0.85	−0.011	**0.93**	−0.016 *
	Text length	**1.00**	−0.424 ***	**1.00**	−0.248 ***	**1.00**	−0.317 ***
	Parallel (yes/no)	0.42	−0.166	0.62	−0.171	0.75	−0.255
**Random effects**	Writing script	0.27		**1.00**		**1.00**	
	Corpus	**1.00**		**1.00**		**1.00**	
	Macro-area	0.00		**0.97**		0.15	
	Country	**1.00**		0.02		0.84	
	Macro-family	**1.00**		**1.00**		**1.00**	
	Sub-family	**1.00**		**1.00**		**1.00**	
	Language	0.39		**1.00**		**1.00**	
**Random slopes**	Macro-area	0.00		0.47		0.04	
	Country	0.28		0.01		0.31	
	Macro-family	0.32		0.29		0.58	
	Sub-family	0.81		**0.95**		**0.91**	

**Table 4:** Spatial autoregressive frequentist model averaging results for morphological complexity as the outcome computed both for the full (*N* = 1443) and the subset version (*N* = 804). Separate models are computed for two different types of weight matrices: $W_{geo}$, which is based on the geographical distances and $W_{phylo}$, which is based on a phylogenetic similarity matrix. Per combination of WALS feature availability and weight matrix type, quantities are computed based on *R* = 35 candidate models. $\sigma_{x}$-values (Equation (7), rounded to two decimal places) reflect the relative importance of each covariate, with higher values indicating a more important variable. Values above 0.90 are shown in bold.$\;{\tilde{\beta }}_{x}$-values (Equation (4), rounded to four decimal places) that pass a two-tailed parametric significance test at *p* < 0.05 are shown in bold. Sig. *** (**) [*] indicates that *p* < 0.001 (*p* < 0.01) [*p* < 0.05].

Version	Full				Subset			
Type of Weight Matrix	$W_{geo}$		$W_{phylo}$		$W_{geo}$		$W_{phylo}$	
Variable	$\sigma_{x}$	${\tilde{\beta }}_{x}$	$\sigma_{x}$	${\tilde{\beta }}_{x}$	$\sigma_{x}$	${\tilde{\beta }}_{x}$	$\sigma_{x}$	${\tilde{\beta }}_{x}$
Vehicularity	**0.98**	−0.1106	**1.00**	−0.0428	**1.00**	−0.1626	**1.00**	−0.1853
Population size	**1.00**	−0.0138 ***	**0.97**	−0.0102 ***	**1.00**	−0.0146 ***	**1.00**	−0.0156 ***
Range size	**1.00**	0.0155 ***	0.00	0.0000	**1.00**	0.0169 ***	**1.00**	0.0210 ***
No. of countries	0.35	0.0022	**1.00**	−0.0144	0.35	−0.0019	0.61	0.0177
Population size x Vehicularity	0.35	0.0030	**0.97**	−0.0004	0.39	0.0030	0.41	0.0039
Range size x Vehicularity	0.28	0.0000	0.00	0.0000	0.48	0.0059	0.42	0.0050
No. of countries x Vehicularity	0.10	−0.0018	0.01	−0.0005	0.10	0.0006	0.19	−0.0034

**Table 5:** Spatial autoregressive frequentist model averaging results for morphological complexity as the outcome computed both for the full (*N* = 1443) and the subset versions (*N* = 804). Separate models are computed for two different types of weight matrices: $W_{geo}$, which is based on the geographical distances and $W_{phylo}$, which is based on a phylogenetic similarity matrix. Per combination of WALS feature availability and weight matrix type, quantities are computed based on *R* = 8695 candidate models. $\sigma_{x}$-values (Equation (7), rounded to two decimal places) reflect the relative importance of each covariate, with higher values indicating a more important variable. Values above 0.90 are shown in bold.${\tilde{\beta }}_{x}$-values (Equation (4), rounded to four decimal places) that pass a two-tailed parametric significance test at *p* < 0.05 are shown in bold. Sig. *** (**) [*] indicates that *p* < 0.001 (*p* < 0.01) [*p* < 0.05].

Version	Full				Subset			
Type of Weight Matrix	$W_{geo}$		$W_{phylo}$		$W_{geo}$		$W_{phylo}$	
Variable	$\sigma_{x}$	${\tilde{\beta }}_{x}$	$\sigma_{x}$	${\tilde{\beta }}_{x}$	$\sigma_{x}$	${\tilde{\beta }}_{x}$	$\sigma_{x}$	${\tilde{\beta }}_{x}$
0—International	0.75	0.0814	0.75	−0.0953	0.75	0.0760	**0.93**	0.1489
1—National	0.87	−0.0225	0.77	0.0624	**0.91**	−0.0929	**0.98**	−0.0987
2—Regional	0.87	−0.3396	0.76	−0.1728	**0.96**	−0.3483	0.81	−0.2544
3—Trade	0.83	−0.1009	0.51	−0.0167	0.81	−0.3084	**0.99**	−0.5257 *
Population size	**1.00**	−0.0144 ***	**1.00**	−0.0115 ***	**1.00**	−0.0147 ***	**1.00**	−0.0166 ***
Range size	**1.00**	0.0155 ***	0.00	−0.0000	**1.00**	0.0170 ***	**1.00**	0.0200 ***
No. of countries	0.55	0.0043	**1.00**	−0.0120	0.52	0.0010	**0.91**	0.0333
Population size x International	0.21	−0.0095	0.19	−0.0007	0.21	−0.0054	0.27	−0.0130
Range size x International	0.20	−0.0043	0.00	0.0000	0.21	−0.0064	0.26	−0.0088
No. of countries x International	0.12	0.0033	0.17	0.0042	0.11	0.0012	0.23	0.0006
Population size x National	0.25	0.0008	0.38	−0.0063	0.23	−0.0011	0.28	−0.0022
Range size x National	0.32	−0.0058	0.00	−0.0000	0.27	0.0027	0.28	0.0013
No. of countries x National	0.14	0.0024	0.35	−0.0148	0.16	0.0054	0.28	0.0082
Population size x Regional	0.59	0.0173	0.50	0.0093	0.38	0.0047	0.33	0.0053
Range size x Regional	0.30	0.0048	0.00	0.0000	0.65	0.0215	0.46	0.0147
No. of countries x Regional	0.14	0.0029	0.18	0.0017	0.14	−0.0013	0.20	−0.0001
Population size x Trade	0.24	0.0008	0.06	0.0002	0.58	0.0172	0.77	0.0279
Range size x Trade	0.28	0.0036	0.00	0.0000	0.30	0.0048	0.36	0.0060
No. of countries x Trade	0.13	0.0004	0.12	−0.0043	0.17	0.0074	0.29	0.0119

## Data Availability

Commented Stata 18 code to reproduce all results is available at https://osf.io/fypx5/ (accessed on 8 November 2024). The original data can be downloaded from https://dx.doi.org/10.6084/m9.figshare.c.4400675 or https://github.com/erc-starfish/koplenig-reply. The updated data on information-theoretic complexity can be downloaded from https://osf.io/xdwjc/ (accessed on 8 November 2024). The data used to generate the phylogenetic and geographic weight matrices are available at https://osf.io/cufv7/ and https://cdstar.eva.mpg.de//bitstreams/EAEA0-B701-6328-C3E3-0/languages_and_dialects_geo.csv (accessed on 8 November 2024). For the 1,000,000 random samples, each consisting of 100 languages selected from the 6880 living languages listed in the Ethnologue (Section 2), I used the Ethnologue Global Dataset (20th edition), a licenced product with restricted terms of use under a personal research licence.

## Appendix A. Eigenvector Spatial Filtering

I first consider linear models of the form:

(A1) y=Fγ+ϵ

where **y** is the *n* × 1 vector morphological complexity values, *n* denotes the number of languages, **F** is a *n* × *q* matrix of *q* eigenvectors, γ is a *n* × 1 vector of parameters and ϵ is the *n* × 1 vector of residuals. Initially, **F** only consists of a *n* × 1 vector of ones for the intercept. The other vectors of **F** are computed based on a transformed version of **W** defined as [79]:

(A2) $$M\equiv \left(I-\frac{11^{Τ}}{n}\right)W\left(I-\frac{11^{Τ}}{n}\right)$$

where **I** is the *n* × *n* identity matrix, **1** is an *n* × *1* vector of ones and Τ denotes the matrix transpose. The eigensystem decomposition of **M** generates *n* eigenvalues and *n* corresponding eigenvectors. The eigenvalues are then sorted in descending order, denoted as $\lambda =(\lambda_{1},\lambda_{2},\lambda_{3},\ldots \lambda_{n})$, so that the largest eigenvalue receives the subscript 1, the second largest eigenvalue receives the subscript 2 and so on. As in the paper, for **W**, I considered $W_{geo}$ and $W_{phylo}$. The corresponding set of eigenvectors can then be denoted as $E_{geo}=(E_{geo,1},E_{geo,2},E_{geo,3},\ldots ,E_{geo,n})$, and $E_{phylo}=(E_{phylo,1},E_{phylo,2},E_{phylo,3},\ldots ,E_{phylo,n})$.

To test for potential autocorrelation among the residuals, I tested the following null hypothesis:

(A3) $$H_{0}:E\left[\epsilon \epsilon^{T}\right]=\sigma^{2}I$$

where **I** is the *n* × *n* identity matrix, and Τ denotes the matrix transpose. To test *H*_0_, I computed the modified version of Moran’s *I* [80], suggested by Kelejian and Prucha [81], based on $W_{geo}$ and $W_{phylo}$. Since *I*^2^ ~ *Χ*^2^(2) [81], I tested *H*_0_ via a standard *Χ*^2^-test with two degrees of freedom. As suggested by [82], for $W_{geo}$ I defined a search set of relevant eigenvectors, $E_{geo}^{*}$, using the following search algorithm, which includes all *x* eigenvectors with a corresponding eigenvalue λ_x_ ≥ (0.1λ_geo,1_); likewise, for $W_{phylo}$. The algorithm [82] then iterates over the eigenvectors in the search set **E^*^**, consisting of $E_{geo}^{*}$ and $E_{phylo}^{*}$. At each step, Equation (A1) is fitted to the data, and the eigenvector **E** that reduces the *Χ*^2^-value the most is selected into **F** and removed from **E^*^**. This iterative procedure is repeated *Χ*^2^(2) < 1, corresponding to a *p*-value of approximately 0.61.

The great advantage is that it is easy to extend Equation (A1):

(A4) y=Fγ+Xβ+ϵ

where, in addition to the above, **X** is a *n* × *p* design matrix of *p* covariates. Here, standard linear least square regression, which is computationally very efficient, provides consistent estimates for β [83] because the identified set **F** of the control variables isolates the spatial and phylogenetic relationships between languages, enabling the construction of models as though the languages were in fact independent [84]. Table A1 summarises the results of the spatial filtering for both the full and the subset versions. Before filtering, there is strong evidence of autocorrelation in the residuals for both versions (both *Χ*^2^-values > 95 at *p* < 0.0001, row 3). *R*^2^ unfiltered (row 4) represents the coefficient of determination before filtering. For the full version, the algorithm identifies a total of 15 eigenvectors, with 12 from $E_{geo}^{*}$ and 3 from $E_{phylo}^{*}$ (row 5). For the subset version, 19 eigenvectors are chosen, 16 from $E_{geo}^{*}$ and 3 from $E_{phylo}^{*}$. After filtering, there is no indication of autocorrelation among the residuals (both *Χ*^2^-values < 1 at *p* > 0.66, row 6). In both cases, the coefficient of determination increases significantly (row 7). In what follows, I used the identified sets for both versions, i.e., **F_full_** and **F_subset_**, as input for Equation (A4).

**Table A1:** Spatial filtering results for both the full and the subset versions.

Version	Full	Subset
***N***	1443	804
***Χ*** **^2^** ** unfiltered**	95.88 (*p* < 0.0001)	269.95 (*p* < 0.0001)
***R*** **^2^** ** unfiltered**	5.45%	9.79%
**Cardinality of F**	*N*_geo_ = 12, *N*_phylo_ = 3	*N*_geo_ = 16, *N*_phylo_ = 3
***Χ*** **^2^** ** filtered**	0.82 (*p* = 0.663)	0.61 (*p* = 0.737)
***R*** **^2^** ** filtered**	18.11%	39.28%

## Appendix B. Bayesian Model Averaging (BMA)

In a BMA, model averaging is conducted in a Bayesian framework [74,75]. For any model *M_j_* of the *R* candidates, we can compute its analytical posterior model probability (PMP) via Bayes theorem [85]:

(A5) $$Ρ\left(M_{j}|D\right)=\frac{Ρ\left(D|M_{j}\right)Ρ(M_{j})}{\sum_{r=1}^{R}Ρ\left(D|M_{r}\right)Ρ(M_{r})}$$

where *D* is the given data and $Ρ(M_{j})$ is an assigned prior. In what follows, I allowed the data to speak for itself by assuming a non-informative prior, i.e., I assumed equal probability for all *R* models [75]. $Ρ\left(M_{j}|D\right)$ can be used to compute a BMA estimator as:

(A6) $${\tilde{\beta }}_{x}=\sum_{j=1}^{R}Ρ\left(M_{j}|D\right)\beta_{x,j}$$

where $\beta_{x,j}$ denotes the estimated effect of variable *x* for model *j*. The posterior inclusion probability (PIP) for predictor variable *x* can be defined as the sum of PMPs that include *x* [75]:

(A7) $${PIP}_{x}=\sum_{j=1}^{R}Ρ\left(M_{j}|D\right)c_{x,j}$$

where $c_{x,j}$ is a binary indicator that is equal to 1 if *x* is explicitly in model *j* and 0 otherwise [21]. The higher ${PIP}_{x}$ the higher its importance. As predictors, I first considered the vehicularity, speaker population size, range size, the number of countries and all higher-order interactions, yielding a total of *R* = 2129 candidates.

As in the main part of the paper, models that include interactions are only considered if the corresponding main effects and all corresponding lower-order interactions are also included in the model. Additionally, note that all quantitative variables are log-transformed and that the results presented in this section and in Appendix C do not change qualitatively if the quantitative predictors are additionally standardised to a mean of 0 and a standard deviation of 2 prior to estimation, as suggested by Gelman (https://statmodeling.stat.columbia.edu/2009/07/11/when_to_standar/, accessed on 8 November 2024).

The identified set of control variables, F_full_ or F_subset_, are always included. (The use of spatial filtering rather than fitting spatial autoregressive models is due to the high computational cost of fitting such non-linear models across a large model space [83]). Table A2 presents the results. For the full version, the only variable with a moderately high PIP of 66.06% is the population size, while all other predictors have PIPs below 25%. Correspondingly, the BMA variable-inclusion summary shows that a model with only population size (and **F_full_**) achieves a PMP of 51.90%. All other models only play a minor role. The results for the subset version are highly comparable, with only population size as an important predictor, with a PIP of 88.52%. The low PIPs for vehicularity again question the linguistic niche hypothesis [1].

I continued by considering a larger model space. As in the main part of the paper, I considered binary indicators for the first four levels of EGIDS (0, 1, 2, 3). Out of curiosity, I have added a fifth EGIDS indicator for EGIDS = 4 (“Literacy in the language is being transmitted through a system of public education”; see Table 1 in the paper). Again, I consider the following three quantitative variables: population size, range size and number of countries. To keep the model space manageable, I only additionally considered possible higher-order correlations between the EGIDS indicators and the first two quantitative variables (population size and range size). In total, the model space consists of *R* = 237,232 models. Table A3 demonstrates that the results are in close agreement with the smaller candidate space: only speaker population size is an important predictor of morphological complexity.

**Table A2:** Bayesian model averaging results for both the full and the subset versions for the small candidate set, with *R* = 2129 considered candidate models. Note that each model in both versions always includes the corresponding set of control variables, F_full_ or F_subset_, identified above (Appendix A). Per version, the first part of the table presents the BMA estimates for each predictor, ${\tilde{\beta }}_{x}$, ranked by their PIP. Predictors with a PIP of less than 1% are omitted. The second part of the table presents results for the top five models ranked by their PMP. ‘x’ in the variable-inclusion summary indicates that the corresponding predictor is included in the model.

Version: Full					
	${\tilde{\beta }}_{x}$	${PIP}_{x}$ **(%)**			
**Population size**	−0.00505	66.06%			
**Vehicularity**	−0.01369	23.34%			
**N countries**	−0.00669	21.18%			
**Range size**	−0.00049	8.35%			
**BMA variable-inclusion summary**					
	**Rank 1 (PMP = 51.90%)**	**Rank 2 (PMP = 12.83%)**	**Rank 3 (PMP = 11.26%)**	**Rank 4 (PMP = 5.79%)**	**Rank 5 (PMP = 4.84%)**
**Population size**	x			x	x
**Vehicularity**		x		x	
**N countries**			x		x
**Version: subset**					
	${\tilde{\beta }}_{x}$	${PIP}_{x}$ **(%)**			
**Population size**	−0.00630	88.52%			
**Range size**	0.00139	17.30%			
**Vehicularity**	−0.00495	12.58%			
**N countries**	−0.00012	5.52%			
**Pop x Range**	−0.00002	1.54%			
**BMA variable-inclusion summary**					
	**Rank 1 (PMP = 65.50%)**	**Rank 2 (PMP = 12.88%)**	**Rank 3 (PMP = 6.72%)**	**Rank 4 (PMP = 3.51%)**	**Rank 5 (PMP = 2.56%)**
**Population size**	x	x		x	
**Range size**		x			
**Vehicularity**			x	x	

**Table A3:** Bayesian model averaging results for both the full and the subset versions for the small candidate set, with *R* = 237,232 considered candidate models. Note that each model in both versions always includes the corresponding set of control variables, F_full_ or F_subset_, identified above (see Appendix A). Per version, the first part of the table presents the BMA estimates for each predictor, ${\tilde{\beta }}_{x}$, ranked by their PIP. Predictors with a PIP of less than 1% are omitted. The second part of the table presents results for the top five models ranked by their PMP. ‘x’ in the variable-inclusion summary indicates that the corresponding predictor is included in the model.

Version: Full						
	${\tilde{\beta }}_{x}$	${PIP}_{x}$ **(%)**				
**Population size**	−0.00570	73.76%				
**N countries**	−0.00842	21.62%				
**EGIDS = 1**	−0.00708	12.15%				
**EGIDS = 4**	−0.00322	8.86%				
**Range size**	−0.00055	8.74%				
**EGIDS = 0**	−0.00861	5.87%				
**EGIDS = 2**	−0.00323	4.46%				
**EGIDS = 3**	−0.00033	2.75%				
**BMA variable-inclusion summary**						
	**Rank 1 (PMP = 49.12%)**	**Rank 2 (PMP = 10.66%)**	**Rank 3 (PMP = 4.46%)**	**Rank 4 (PMP = 4.32%)**	**Rank 5 (PMP = 3.61%)**	
**Population size**	x		x	x	x	
**EGIDS = 1**				x		
**EGIDS = 4**					x	
**N countries**		x	x			
**Version: subset**						
	${\tilde{\beta }}_{x}$	${PIP}_{x}$ **(%)**				
**Population size**	−0.00574	82.43%				
**EGIDS = 1**	−0.01421	23.17%				
**Range size**	0.00111	14.13%				
**EGIDS = 0**	−0.01684	11.36%				
**EGIDS = 4**	−0.00353	7.63%				
**EGIDS = 3**	−0.00060	4.24%				
**EGIDS = 2**	−0.00153	3.99%				
**N countries**	−0.00057	3.97%				
**BMA variable-inclusion summary**						
	**Rank 1 (PMP = 47.90%)**	**Rank 2 (PMP = 7.46%)**	**Rank 3 (PMP = 6.25%)**	**Rank 4 (PMP = 5.83%)**	**Rank 5 (PMP = 3.65%)**	
**Population size**	x	x		x	x	
**Range size**		x				
**EGIDS = 0**					x	
**EGIDS = 1**			x	x		

## Appendix C. Non-Parametric Permutation Testing

To test in a non-parametric setting, we write:

(A8) y=Zγ+Xβ+ϵ

where, in addition to the above, Z is a matrix of nuisance/control variables. The predictor of interest, represented by X, is either vehicularity or population size. In what follows, I used a generic variant of the *Freedman-Lane* permutation testing procedure that does not make any assumptions about the mechanism that generated the data [58,86]

1. Let *y* denote the morphological complexity variable. Per version (full/subset), *y* is regressed onto the predictor of interest and the control variables. This model is used to calculate the test statistic *T*_0_, which is the *t*-statistic of the estimated parameter for vehicularity or population size.
2. Further, *y* is regressed only onto the control variables in a reduced model, and fitted values $\hat{y}$ and residuals $\hat{\varepsilon }$ are obtained. This means that $\hat{y}$ contains the sample mean plus contributions based on the control variables.
3. The residuals $\hat{\varepsilon }$ are randomly permuted, called the resulting variable ${\hat{\varepsilon }}^{*}$, and a new variable is computed that is defined as the sum of the fitted values and the randomly permuted residuals, i.e., $y^{*}=\hat{y}+$ ${\hat{\varepsilon }}^{*}$.
4. $y^{*}$ is regressed onto the predictor of interest and the control variables in order to calculate the test statistic of interest and call this statistic $T_{j}^{*}$.
5. Steps c and d are repeated 10,000 times to calculate the reference distribution of $T^{*}$.
6. Count the number of times where ${|T}_{j}^{*}|\geq |T_{o}|$ and divide that number by 10,000. The result is the *p*-value.

The intuitive idea behind this permutation test is that if the null hypothesis holds—meaning the predictor of interest is unrelated to morphological complexity—then the derived datasets, i.e., those with randomly permuted residuals, should be indistinguishable from the original dataset. As Freedman and Lane [58] described it: “a small reported significance level indicates an unusual data set”. Table A4 presents the results. Vehicularity is only a significant predictor of morphological complexity when speaker population size is not included as a control. Conversely, population size remains a significant predictor of morphological complexity regardless of the set of control variables.

**Table A4:** Results of the permutation test. For each version (full/subset), morphological complexity is regressed onto the predictor of interest and the control variables. F_full_ or F_subset_ represents the set of control variables, which are identified above (Appendix A). The fourth column shows the estimated effect and the *t*-value in brackets. The last column shows the permutation-based *p*-value. Sig. *** (**) [*] indicates that *p* < 0.001 (*p* < 0.01) [*p* < 0.05].

Version	Predictor of Interest	Control Variables	${\hat{\beta }}_{x}(t)$	*p_perm_*
full	Vehicularity	**F_full_**	−0.071 (−3.415) ***	0.0000
		Population size + **F_full_**	−0.041 (−1.691)	0.1000
subset		**F_subset_**	−0.052 (−2.971) **	0.0022
		Population size + **F_subset_**	−0.023 (−1.082)	0.2786
full	Population size	**F_full_**	−0.008 (−3.801) ***	0.0000
		Vehicularity + **F_full_**	−0.006 (−2.373) *	0.0192
subset		**F_subset_**	−0.007 (−3.652) ***	0.0004
		Vehicularity + **F_subset_**	−0.005 (−2.373) *	0.0178
